# Construction Notes

**Published:** 1896-05-02

**Authors:** 


### CONSTRUCTION NOTES.

### Bearsden Convalescent Home.

The Shaw Convalescent Home at Bearsden has been formally handed over to the directors of the Glasgow Royal Infirmary. This home, which is perfect in all its equipment, was raised to the memory of Mr. Richard Shaw by his sister, for which estimable object she set aside the sum of £40,000 to carry out her purpose. The new building, standing in the centre of its own ground of twelve acres, occupies an elevated and commanding position at the further end of the village of Bearsden. Accommodation is here provided for fifty patients, but the number may be increased on emergency. The main front building, not including the basement, is three storeys in height and 176 feet long. It is treated architecturally in free modern style of Gothic, the centre being adorned with a lofty square tower. There are day and recreation rooms on the ground floor, with staircases leading to dormitories on the upper floor, and direct communication is given on each side from the male and female departments to the dining hall. The basement contains a smoking room and a large workroom for the women.

### Dundee Infirmary Nurses' Home.

Through the munificence of Mr. W. Ogilvy Dalgleish, of Errol Park, the Dundee Royal Infirmary directors are about to erect a home for the nurses identified with the establishment, while important structural alterations are also contemplated. This building will be an attractive one, and the site fixed for it is immediately to the north of the new operation theatre just completed. In the form of the letter "L" and three storeys in height, it will have a southern frontage of ninety-five feet. On the ground floor there is to be a drawing-room, a sister's sitting-room, a library, cloak-room and lavatory, small kitchen and scullery where tea can be made, six dormitories with bath-room, lavatory, &c. On the second floor there will be sixteen dormitories, with two bath-rooms, lavatories, &c., also head sister's sitting-room, while on the third floor there will be fifteen dormitories with two bath-rooms, lavatories, &c., also sick-room. None of the dormitories are less than twelve feet, and all are heated by steam pipes, and ventilated at the floors and ceilings. The public rooms have fire-places, and are also heated by hot-water pipes. These elevations, though perhaps not very ornamental, are in the character of the adjoining infirmary. When the nurses' home is completed, the space in the principal building at present required for the nurses will be available for the accommodation of patients. The architect for this home is Mr. A. Johnstone.

